# The Maternal Microbiome Programs the m^6^A Epitranscriptome of the Mouse Fetal Brain and Intestine

**DOI:** 10.3389/fcell.2022.882994

**Published:** 2022-07-07

**Authors:** Zhuoyu Xiao, Sun Liu, Zengguang Li, Jinru Cui, Hailan Wang, Zihan Wang, Qihuan Ren, Laixin Xia, Zhijian Wang, Yuan Li

**Affiliations:** ^1^ Department of Developmental Biology, School of Basic Medical Sciences, Southern Medical University, Guangzhou, China; ^2^ Department of Obstetrics and Gynecology, Nanfang Hospital, Southern Medical University, Guangzhou, China

**Keywords:** maternal microbiome, m^6^A, fetal development, Wnt signaling pathway, METTL3

## Abstract

The microbiome exerts profound effects on fetal development and health, yet the mechanisms underlying remain elusive. N6-methyladenosine (m^6^A) plays important roles in developmental regulation. Although it has been shown that the microbiome affects the mRNA m^6^A modification of the host, it remains unclear whether the maternal microbiome affects m^6^A epitranscriptome of the fetus so as to impact fetal development. Here, we found that loss of the maternal microbiome altered the expression of m^6^A writers and erasers, as well as the m^6^A methylome of the mouse fetal brain and intestine on embryonic day 18. From the m^6^A profiles, we identified 2,655 and 2,252 m^6^A modifications regulated by the maternal microbiome in the fetal brain and intestine, respectively, and we demonstrated that these m^6^A-modified genes were enriched in the neuro/intestinal developmental pathways, such as the Wnt signaling pathway. Finally, we verified that antibiotic treatment mostly recapitulated changes in m^6^A, and we further showed that the loss of heterozygosity of *Mettl3* rescued m^6^A levels and the expression changes of some developmental genes in the fetal intestine that resulted from antibiotic treatment. Collectively, our data revealed that the maternal microbiome programs the m^6^A epitranscriptome of the mouse fetal brain and intestine.

## Introduction

It is universally recognized that the microbiome exerts profound effects on host physiology and health, including host metabolism, circadian rhythm, intestinal morphology, and the development of the nervous system (Zhang et al., 2020; Brooks et al., 2021; Seki et al., 2021; Willyard, 2021; Wu J. et al., 2021; Wu Q. et al., 2021). Emerging studies have revealed that loss of the maternal microbiome impairs axonogenesis (Vuong et al., 2020), and that maternal exposure to antibiotics contributes to gut dysbiosis, immune dysfunction, and the occurrence of inflammatory bowel disease (IBD) in offspring (Miyoshi et al., 2017). These findings indicated that loss of the maternal microbiome impairs the fetal development and neonatal health in early life. However, the mechanisms underlying the actions of the maternal microbiome on the fetus remain elusive.

N6-methyladenosine (m^6^A) has been shown to be the most abundant and a highly conserved modification on messenger RNAs (mRNAs) and lncRNAs in mammals (Dominissini et al., 2012; Zhao et al., 2017a; Liu et al., 2020; Shu et al., 2020; Deng et al., 2021). mRNA m^6^A possesses a consensus motif of RRACH (R denoting G or A, and H reflecting A, C, or U) and it is principally found at stop codons, 3´untranslated regions (3´UTRs), and long exons (Dominissini et al., 2012; Roundtree et al., 2017). m^6^A is produced by the METTL3–METTL14 core methyltransferase complex (Liu et al., 2014; Liu X. et al., 2021), erased by demethylases FTO and ALKBH5 (Jia et al., 2011; Zheng et al., 2013; Roundtree et al., 2017), and recognized by readers such as the YTH family proteins (Dominissini et al., 2012; Wang X. et al., 2014; Alarcón et al., 2015). m^6^A regulates various physiological processes, such as RNA stability (Dominissini et al., 2012; Wang X. et al., 2014; Alarcón et al., 2015; Huang et al., 2018), splicing (Xiao et al., 2016; Ke et al., 2017), translation (Meyer et al., 2015; Wang et al., 2015; Zhou et al., 2015; Shi et al., 2017), and signaling pathways (Li H.-B. et al., 2017; Huang H. et al., 2019; Uddin et al., 2021). Also, it occupies important roles in stem cell self-renewal (Li et al., 2018; Liu J. et al., 2021), embryonic development (Batista et al., 2014; Wang Y. et al., 2014; Chen et al., 2015; Geula et al., 2015; Vu et al., 2017; Bertero et al., 2018), tissue development (Zheng et al., 2013; Li H.-B. et al., 2017; Yoon et al., 2017; Zhao et al., 2017b; Wang et al., 2018), tumorigenesis (Ma et al., 2016; Li Z et al., 2017; Su et al., 2018; Huang Y et al., 2019; Su et al., 2020; Chen et al., 2021), and the progression of other human diseases (Fischer et al., 2009; Church et al., 2010; Mathiyalagan et al., 2019). It has been shown that the microbiome affects the mRNA m^6^A modification on the host tissues, especially the brain, intestine, and liver (Wang et al., 2019; Jabs et al., 2020). However, it remains unclear whether the maternal microbiome affects m^6^A epitranscriptome of the fetal brain, intestine, and liver so as to impact fetal development.

Herein, we demonstrated that the expression of m^6^A writers and erasers in the brain and intestine of the mouse fetus is altered by the maternal microbiome. Using MeRIP-seq, we systematically investigated the transcriptome-wide m^6^A methylome profiles of the mouse fetal brain and intestine, and we discovered that the maternal microbiome programs the fetal m^6^A methylome, and that m^6^A-modified genes regulated by the maternal microbiome are enriched in fetal neuro/intestine developmental pathways, such as the Wnt signaling. More importantly, antibiotic treatment recapitulated m^6^A alterations in the mouse fetal intestine and brain, and loss of heterozygosity of *Mettl3* rescued this effect. Our findings collectively indicate that the maternal microbiome programs the m^6^A epitranscriptome of the mouse fetal brain and intestine, and this may provide a promising basis to explore the mechanisms by which the maternal microbiome influences fetal development and diseases.

## Materials and Methods

### Fetal Tissues Collection From SPF and GF Mice

Specific pathogen-free (SPF) pregnant mice (*n* = 3) and germ-free (GF) pregnant mice (*n* = 3) purchased from GemPharmatech Co., Ltd. were dissected on embryonic day 18 (E18), and the fetal tissues (brain, intestine, and liver) were collected and stored at −80°C for subsequent analyses.

### PCR Amplification and qPCR Analysis of 16S rRNA Genes

A total of 40 mg mouse fecal pellets were suspended in 200 μl lysis buffer (5 mM EDTA, 0.2% SDS, 0.2M NaCl, and 0.1M Tris-HCl) supplemented with 4 μl of 20 mg/ml proteinase K. The mixtures were disrupted with a grinding rod and then incubated at 56 °C for 6 h. After centrifugation, the supernatant was used for 16S rRNA gene amplification, and the PCR products were visualized on a 2% agarose gel stained with ethidium bromide under UV light. The supernatant from CONV and ABX mice was used for the 16S rRNA gene qPCR analysis. The 16S rRNA gene was detected using two sets of universal bacterial primers: 27F and 1492R; 8F and 1541R. The primers are listed in Supplementary Table S1.

### Tissues Lysate Preparation and Western Blots

Frozen tissues were homogenized and lysed in RIPA buffer (50 mM Tris-HCl pH 7.5, 1% Nonidet P-40, 0.5% sodium deoxycholate, 0.05% SDS, 1 mM EDTA, and 150 mM NaCl) with freshly added phosphorylase inhibitors and protease inhibitors, and then centrifuged for 20 min at 13,000 × g. The supernatant was aspirated and loaded for the Western immunoblotting analysis. The following antibodies are used: METTL3 (A8370, Abclonal, 1:1,000), METTL14 (HPA038002, Sigma-Aldrich, 1:1,000), FTO (27226-1-AP, Proteintech, 1:1,000), ALKBH5 (16837-1-AP, Proteintech, 1:1,000), and β-actin (66009-1-Ig, Proteintech, 1:5,000).

### RNA Isolation and mRNA Purification

Fetal mouse tissues were homogenized in 1 ml of TRNzol Universal Reagent (TIANGEN) with glass beads using a LUKYM-I homogenizer, and total RNA was isolated following the manufacturer’s protocol. mRNA was separated from total RNA using a Dynabeads mRNA purification kit (Thermo Fisher Scientific), with two rounds of purification.

### LC-MS/MS Quantification of m^6^A mRNA Modification

LC-MS/MS was performed essentially as described previously (Li et al., 2020). In brief, purified mRNA was digested to nucleosides by nuclease P1 and CIAP, and then it was diluted to 10 ng/μl using nuclease-free water. The samples were filtered and injected into an Agilent Poroshell 120 column coupled online to an AB SCIEX Triple Quad 5500 LC mass spectrometer (Applied Biosystems) in a positive electrospray ionization mode. Concentrations of m^6^A and A were determined based on standard curves of the nucleosides, and the m^6^A/A ratio was calculated.

### RT-qPCR

Total RNA (5 μg) from fetal mouse tissues was reverse-transcribed using a GoScript Reverse Transcription System (Promega), and quantitative real-time PCR was executed using a 2 × RealStar Green Power Mixture (GenStar). The fluorescence intensity of the amplification process was monitored using a LightCycler96 system (Roche). The primers are listed in Supplementary Table S2.

### Methylated RNA Immunoprecipitation Sequencing

MeRIP experiments were executed as previously reported (Xiao et al., 2019). In brief, approximately 90 µg of total RNA was fragmented into 100- to 300-nucleotide (nt)-long fragments by zinc acetate, followed by the addition of EDTA to terminate the reaction. Then, 5 µg of fragmented RNA was taken as the input control and the remainder was incubated with m^6^A antibodies (4 μg, Abcam, ab151230) in IP buffer (150 mM NaCl, 0.05% NP-40, and 10 mM Tris-HCl) containing RNase inhibitor (Promega), and the mixture was subsequently bound to wash Dynabeads protein G (Invitrogen). After stringent wash, the m^6^A-containing fragments were eluted by competition with 1 mg/ml N6-methyladenosine (Selleck Chemicals). Both the immunoprecipitated RNA fragments and the input RNA were ultimately extracted for library construction using a SMARTer Stranded Total RNA-Seq Kit v2 - Pico Input Mammalian (Takara) following the manufacturer’s protocol. We then performed sequencing using an Illumina Nova platform.

### MeRIP-Seq Data Processing and Mapping

Prior to mapping, all raw data were filtered to remove adapters, and low-quality reads using Trimmomatic (Bolger et al., 2014). Reads of all samples that mapped to rRNA FASTA sequences from UCSC gene annotation (mm10) using bowtie2 (Langmead and Salzberg, 2012) were discarded, and the remaining reads were aligned to the mouse reference genome (GRCm38) using HISAT2 (Pertea et al., 2016). Then mapped files were filtered to keep unique and high mapping quality reads for further analysis using Picard and SAMtools (Li et al., 2009).

### m^6^A Peak Calling

m^6^A peaks were identified using MeTPeak. A custom transcriptome annotation file, assembled by StringTie (Pertea et al., 2016) using all sample reads, was created to include intronic and intergenic m^6^A peaks. All other parameters were set to the default settings. The annotatePeaks.pl script from the Homer software suite (Heinz et al., 2010) was used for m^6^A peak annotation.

### Evaluation of the Similarity of m^6^A Between Samples

m^6^A peaks identified in all samples were merged, and featureCounts (Liao et al., 2014) was used to count the fragments that were mapped to the merged peaks. The normalized fragment counts of each peak in MeRIP-seq (MFPKM) were calculated using (methylated fragment counts mapped to the peak × 10^9^)/(length of the peak × total counts of the mapped fragment), and the normalized fragment counts of each peak in input-seq (IFPKM) were calculated using (input fragment counts mapped to the peak × 10^9^)/(length of the peak × total counts of mapped fragments). The methylation level was then calculated for each peak by dividing the MFPKM by the IFPKM. The Pearson correlation coefficient of log2-scaled m^6^A levels across all samples was calculated using corrplot to represent the similarity of each sample.

### Determination of m^6^A Motif and Distribution Pattern

m^6^A peaks were used for motif search using the findMotifsGenome.pl script from the Homer software suite, using “-rna” and “-len 5” parameters. The R package Guitar (Cui et al., 2016) was used to analyze and plot the distribution of m^6^A on mRNA.

### Identification of Differentially Methylated Regions

The regions in which the GF group mean m^6^A level was 1.5 fold higher than the SPF group mean m^6^A level were defined as GF group up regions. Also, the regions in which the GF group mean m^6^A level was 1.5 fold lower than the SPF group mean m^6^A level were defined as GF group down regions.

### Gene Ontology Analysis of Differential m^6^A-Methylated Genes

Differentially methylated regions were assigned to mouse genes using the annotatePeaks.pl script from the Homer software suite. The gene list was used for pathways and GO term enrichment using the clusterProfiler (Wu T. et al., 2021).

### MeRIP-qPCR

The input RNA and the immunoprecipitated RNA fragments from mouse fetal tissues were reverse-transcribed using a GoScript Reverse Transcription System (Promega), and then they were analyzed using real-time qPCR. The ratio of immunoprecipitated RNA to the input of each peak was calculated and normalized to GAPDH. The primers are listed in Supplementary Table S3.

### Cell Culture and Cell Line Generation

Mouse embryonic stem cell line E14TG2a (mES cells) was cultured with the N2B27 base medium supplemented with 1 mM glutamine (Invitrogen), 1% nonessential amino acids (Invitrogen), 0.15 mM 1-thioglycerol (Sigma), 100 U/ml of penicillin–streptomycin (Invitrogen), 25 μg/ml of BSA (Sigma), 1 μM MEK inhibitor PD0325901 (Selleck Chemicals), 3 μM GSK3β inhibitor CHIR99021 (Selleck Chemicals), 2% KOSR (Thermo Fisher), and 1000 U/ml of ESGRO leukemia inhibitory factor LIF (Millipore) on plates coated with 0.2% gelatin.

### *Mettl3* ^–/–^ mES Cell Line Generation

*The Mettl3* ^–/–^ mES cell line was generated using CRISPR-Cas9 as described previously (Shalem et al., 2014) and the sgRNA sequences are shown in Supplementary Table S4. In brief, sgRNAs were designed on http://crispr-era.stanford.edu/and cloned into the pXPR_001 plasmid. Then, pXPR_001 plasmid was transfected into mES cells using Lipofectamine 3000 (Invitrogen, L3000015). After 12 h, 3 μg/ml of puromycin was added and resistant cells were plated for single colony isolation. Colonies with the desired mutation were identified by Sanger sequencing.

### RNA Stability Assay

mES cells cultured in 12-well plates at 70–80% confluency were treated with actinomycin D (5 μg/ml final concentration, MCE, HY-17559) for 0, 2, 4, and 8 h before being collected for the extraction of total RNA. RNA was then reverse-transcribed using GoScript Reverse Transcriptase (Promega), and analyzed using real-time qPCR. Expression levels of RNA were calculated and normalized to *GAPDH* first, and then to the 0 h time point. The mRNA stability of genes was estimated by the half-life of mRNA and calculated using GraphPad Prism 5.0. The primers are listed in Supplementary Table S3.

### Animals

All of the mice were group-housed in a temperature-controlled (22 ± 1 °C) room with a 12:12-h light:dark cycle, and they had free access to food and water. *Mettl3* ^*flox/+*^ mice were generated by Cyagen by inserting loxP sites with the same direction on both sides of exons 2 and 3 of the *Mettl3* gene. Male *Mettl3* ^flox/+^ mice were crossed with female *Mettl3* ^flox/+^ mice to obtain *Mettl3* ^flox/flox^ mice. Next, *Mettl3* ^flox/flox^ mice were first crossed with DDX4-Cre mice to generate *Mettl3* ^flox/+^; DDX4-Cre mice, and the latter were then crossed with wild-type mice to generate *Mettl3* ^−/+^ heterozygous mice. The genotype of each mouse was determined using the genomic DNA extracted from tail tissue.

### Antibiotic Treatment of Mice

To mimic GF status, conventional mice (CONV) were treated with antibiotics (ABX), based on methods previously described (Vuong et al., 2020). In brief, 10- to 12 -weeks-old female mice were provided with a mixture of four antibiotics (vancomycin 0.5 g/L, neomycin 1 g/L, ampicillin 1 g/L, and amphotericin-B 0.1 g/L) in their water for 1 week. Female mice were then paired with male mice and gestational day 0.5 was determined by observation of a copulatory plug. Pregnant mice (*n* = 3) were maintained on ABX in their drinking water until embryonic day 18 (E18), and then dissected to obtain fetal tissues (brain and intestine).

### Statistical Analysis

We expressed our measurement data as mean ± SEM. *T* tests were used for comparisons between two groups. Significant differences were represented by asterisks as follows: *p < 0.05, **p < 0.01, and ***p < 0.001, and ns, not significant.

## Results

### Loss of the Maternal Microbiome Alters the Expression of m^6^A Writers and Erasers in the Fetal Brain and Intestine

**FIGURE 1:**
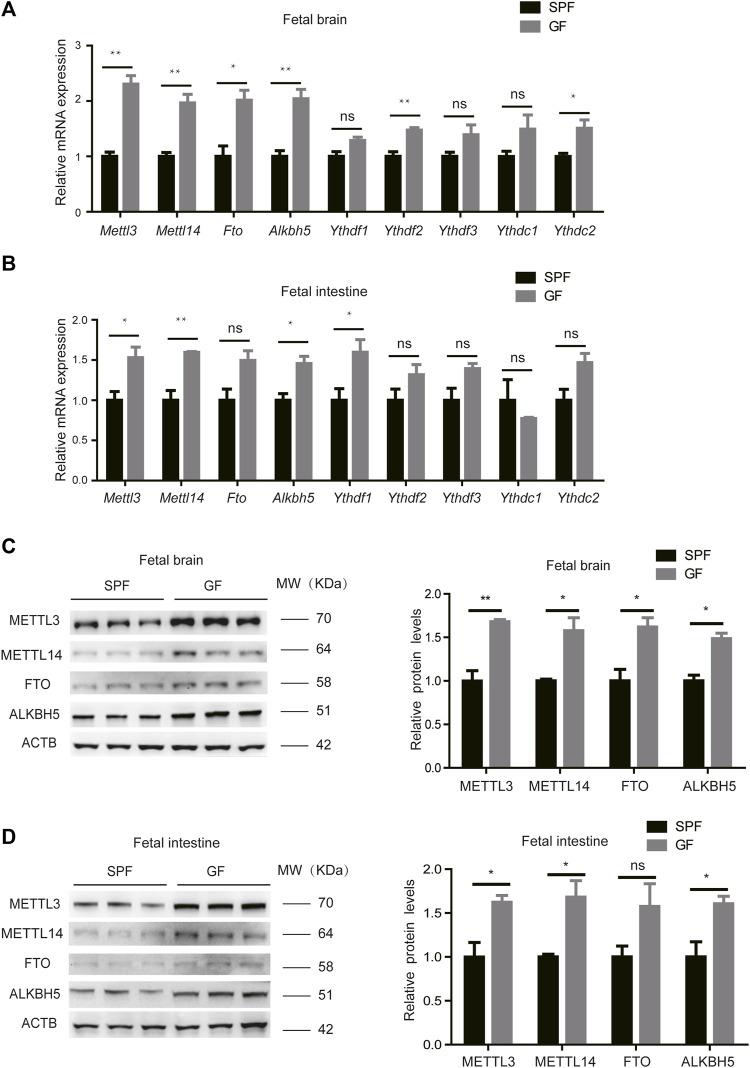
Loss of the maternal microbiome alters the expression of m^6^A writers and erasers in fetal mice. **(A,B)** Relative mRNA expression levels of m^6^A writers, erasers, and readers in fetal brains **(A)** and intestines **(B)** of SPF and GF mice. **(C,D)** Western blots showing the protein expression of m^6^A writers and erasers in fetal brains **(C)** and intestines **(D)** of SPF and GF mice, and relative protein expression levels were calculated based on the band density in Western blotting results.

We initially collected fecal pellets from germ-free (GF, *n* = 3) and specific pathogen-free (SPF, *n* = 3) pregnant mice, and the absence of intestinal microbiota in the GF mice was confirmed by 16S rRNA gene amplification (Supplementary Figure S1A). We, then, examined the levels of m^6^A regulators in the mouse fetal brain, intestine, and liver, including writers (METTL3 and METTL14), erasers (FTO and ALKBH5), and readers (YTH-domain family proteins). Using RT-qPCR, we determined that mRNA levels of m^6^A writers and erasers are highly expressed in the fetal brain and intestine from GF pregnant mice (hereafter designated GFB and GFI, respectively) compared to the corresponding tissues from SPF pregnant mice (hereafter designated SPFB and SPFI, respectively). However, the differences in m^6^A reader expression levels are much less marked (Figures 1A,B). Nevertheless, the expression of these proteins is similar in the fetal livers of these two types of mice (Supplementary Figure S1B). A similar tendency in the alteration of protein expression is also uncovered using the Western blotting analysis (Figures 1C,D). Taken together, these results indicated that loss of the maternal microbiome alters the expression of m^6^A writers and erasers in the fetal brain and intestine.

### m^6^A Modification Profiles in the Fetal Brain and Intestine

**FIGURE 2:**
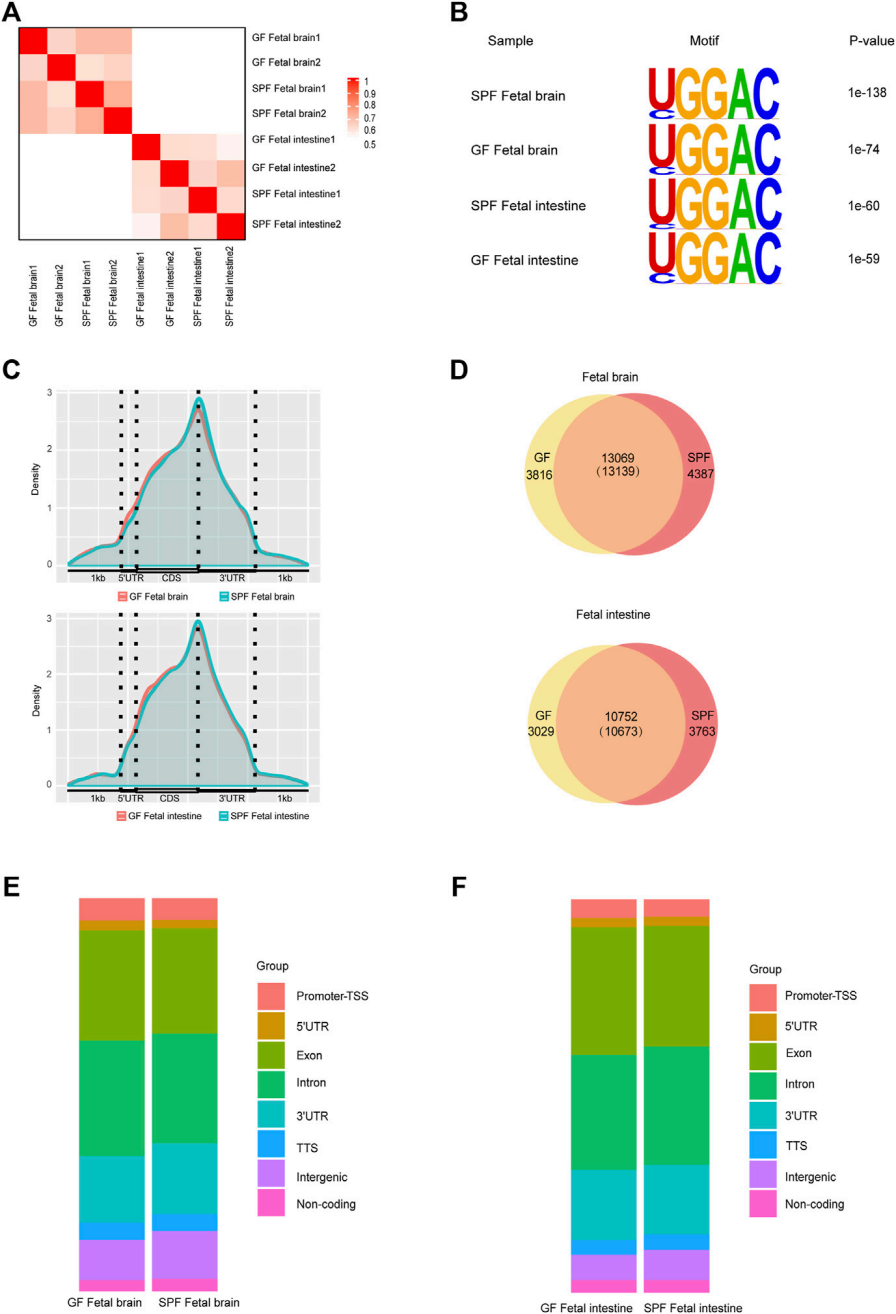
Modification profiles of m^6^A in the fetal brain and intestine. **(A)** Similarity (using Pearson’s correlation) of m^6^A peaks between each pair of samples. **(B)** Motif analysis of m^6^A peaks in fetal brains and intestines of SPF and GF mice. **(C)** Pattern distribution of m^6^A across the mRNA regions in the fetal brain and intestine. m^6^A peaks were mapped back to the corresponding genes, and assigned as originating from the 5′-UTR, coding sequence (CDS), or 3′-UTR. **(D)** Venn diagram showing the overlap of m^6^A peaks between fetal brains and intestines of SPF and GF mice. **(E,F)** Bar charts showing the distribution of m^6^A peaks in the fetal brain **(E)** and intestine **(F)**.

To further investigate whether the maternal microbiome participates in modulating the m^6^A epitranscriptome of offspring, we first detected total m^6^A levels of mouse fetal tissues. We did not observe an apparent change in the global mRNA m^6^A levels between SPF and GF mice as revealed by LC-MS/MS (Supplementary Figure S2A). We, thus, characterized m^6^A methylomes of both mouse fetal brain and intestine (SPFB and GFB and SPFI and GFI—using two independent biological replicates for both) by an m^6^A-immuno-coprecipitation sequencing (MeRIP-seq) analysis. The samples of the same tissue type were clustered well (Figure 2A) and the classic GGAC motif was observed in the fetal brain and intestine (Figure 2B). In agreement with previous studies (Dominissini et al., 2012; Roundtree et al., 2017), the distribution of m^6^A signals around mRNA in the two types of fetal tissue samples was mostly presented in the CDS and 3’UTR, and to a lesser extent in the 5’UTR (Figure 2C). We identified the numbers of m^6^A peaks from these fetal tissues (17,526 in SPFB, 16,885 in GFB, 14,436 in SPFI, and 13,781 in GFI), and we ascertained that approximately three-fourths of the m^6^A peaks overlapped in both fetal brain and intestine (Figure 2D). Compared with SPFB, GFB showed some changes in patterns of m^6^A peaks, with a relative elevation in exonic (SPF 26.81% vs. GFB 28%) and intronic regions (SPF 27.86% vs. GFB 29.38%), and a relative diminution in the 3’untranslated region (3’UTR) from 18 to 16.96% (Figure 2E). Compared with SPFI, the GFI also showed some alterations in patterns of m^6^A peaks with a relative augmentation in exonic regions (SPFI 30.68% vs. GFI 32.5%), and a relative reduction in intronic regions from 30.12 to 29.18% and intergenic regions of 7.7–6.41% (Figure 2F).

### The Maternal Microbiome Regulates the m^6^A of Neurodevelopment Genes in the Mouse Fetal Brain

**FIGURE 3:**
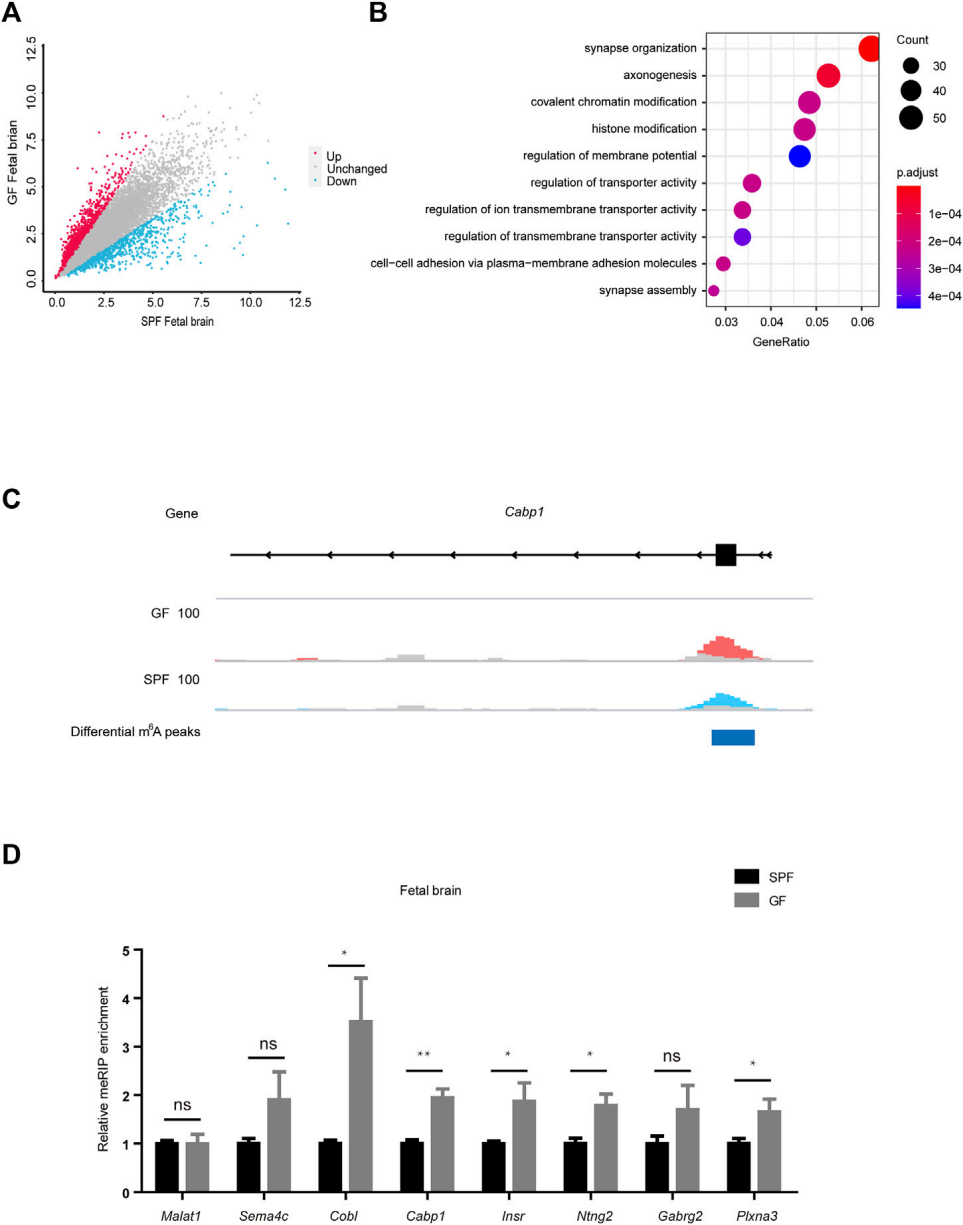
Maternal microbiome regulates the m^6^A of neurodevelopment genes in the mouse fetal brain. **(A)** Scatter diagram shows the number of differential m^6^A peaks in the GF fetal brain. **(B)** Gene ontology-enrichment analysis of genes containing upregulated m^6^A peaks in the GF fetal brain. **(C)** Integrated genome viewer (IGV) shows the distribution of representative differential m^6^A peaks in *Cabp1*. GF IP, SPF IP, and input are shown in red, blue, and gray, respectively. **(D)** Validation of the relative m^6^A enrichments of *Sema4c*, *Cobl*, *Cabp1*, *Insr*, *Ntng2*, *Gabrg2*, and *Plxna3* in SPF and GF fetal brains by m^6^A-immunoprecipitation (IP)-qPCR.

To investigate the dynamic characteristics of m^6^A methylation, we further analyzed the differential m^6^A peaks in mouse fetal tissues. As shown in Figure 3A, GFB manifested 2072 upregulated m^6^A peaks and 583 downregulated m^6^A peaks (with the criterion of fold-change ≥1.5). In further examination of the genomic distribution in all three mRNA regions of differential m^6^A peaks, we demonstrated that a majority of the differential m^6^A peaks were in CDS and 3’UTR (Supplementary Figure S3A). Mapping these reads of differential m^6^A peaks to the genome, we identified 1147 genes with upregulated m^6^A peaks and 496 genes with downregulated m^6^A peaks (Supplementary Figure S3B). To further study the biological significance of dysregulated m^6^A modifications in the fetal brain, we conducted GO analyses of differentially m^6^A-methylated genes (Figure 3B and Supplementary Figure S3C). We concentrated on the function of m^6^A-hypermethylated genes and showed that these genes were significantly enriched in pathways related to neurodevelopment, such as synapse formation and axonogenesis. The read coverage plot of a representative gene *Cabp1* associated with neurodevelopment was depicted in Figure 3C, and the m^6^A levels of genes (*Sema4c*, *Cobl*, *Cabp1*, *Insr*, *Ntng2*, *Gabrg2*, and *Plxna3*) were increased in GFB as revealed by using the MeRIP-qPCR analysis (Figure 3D). In addition, the transcript levels of these genes were confirmed by using the RT-qPCR analysis (Supplementary Figure S3D). Collectively, these data suggest that the maternal microbiome regulates the m^6^A of neurodevelopment genes in the mouse fetal brain.

### The Maternal Microbiome Regulates Fetal Intestinal m^6^A-Modified Genes in the Wnt Signaling Pathway

**FIGURE 4:**
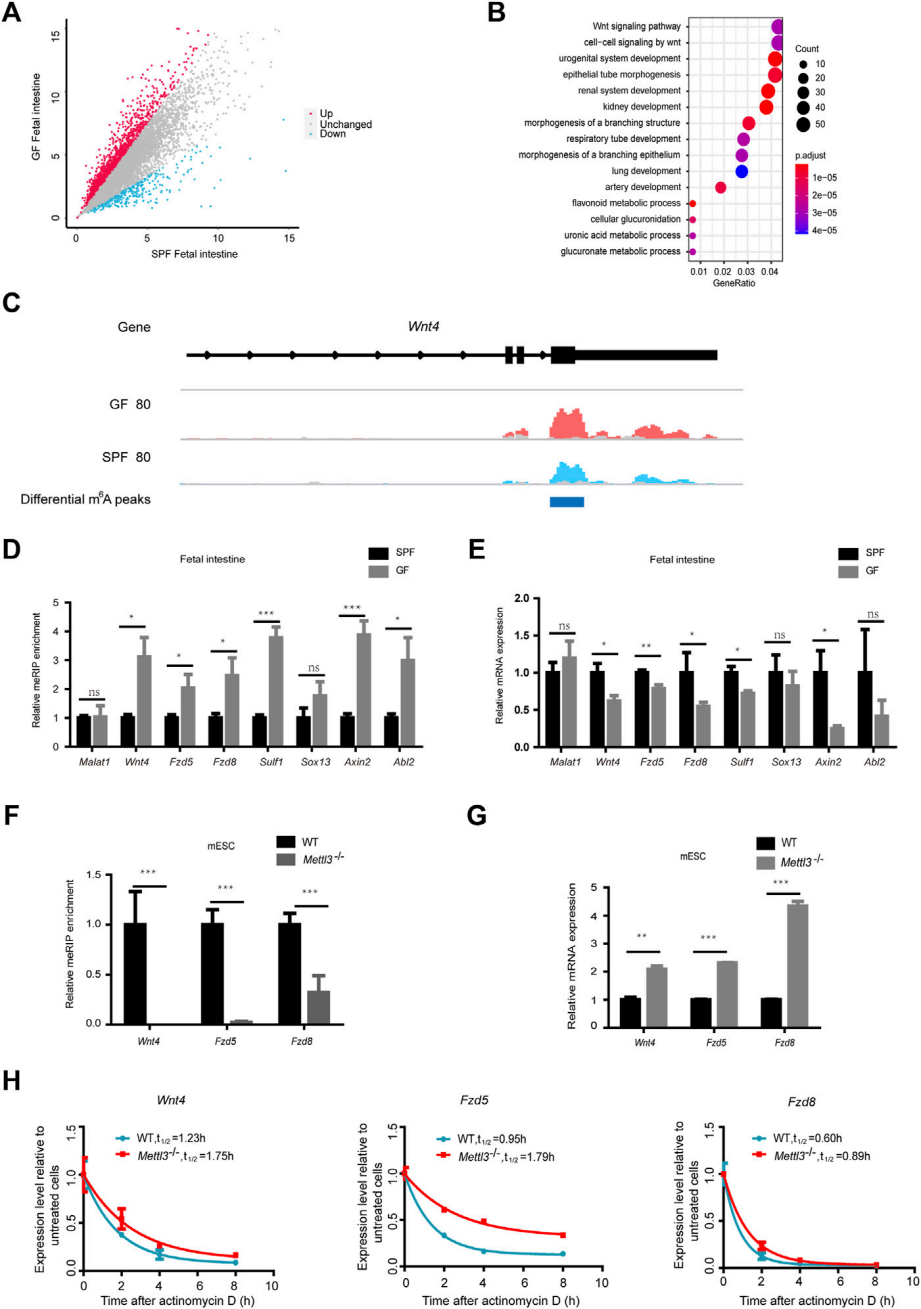
Maternal microbiome regulates fetal intestinal m^6^A-modified genes in the Wnt signaling pathway. **(A)** Scatter diagram shows the number of differential m^6^A peaks in the GF fetal intestine. **(B)** Gene ontology-enrichment analysis of genes containing upregulated m^6^A peaks in the GF fetal intestine. **(C)** IGV shows the distribution of representative differential m^6^A peaks in *Wnt4.* GF IP, SPF IP, and input are shown in red, blue, and gray, respectively. **(D)** Validation of the relative m^6^A enrichments of *Wnt4*, *Fzd5*, *Fzd8*, *Sulf1*, *Sox13*, *Axin2*, and *Abl2* in SPF and GF fetal intestines by m^6^A-immunoprecipitation (IP)-qPCR. **(E)** Validation of the relative mRNA expression levels of *Wnt4*, *Fzd5*, *Fzd8*, *Sulf1*, *Sox13*, *Axin2*, and *Abl2* in SPF and GF fetal intestines. **(F)** Validation of the relative m^6^A enrichments of *Wnt4*, *Fzd5*, and *Fzd8* in the WT and *Mettl3* ^-/-^ mouse embryonic stem cell line E14TG2a by m^6^A-immunoprecipitation (IP)-qPCR. **(G)** Validation of the relative mRNA expression levels of *Wnt4*, *Fzd5*, and *Fzd8* in the WT and *Mettl3* ^-/-^ mouse embryonic stem cell line E14TG2a. **(H)** Half-life (t_1/2_) of *Wnt4*, *Fzd5*, and *Fzd8* mRNA in the WT and *Mettl3* ^-/-^ mouse embryonic stem cell line E14TG2a after actinomycin D treatment.

As shown in Figure 4A, GFI reflected 2068 upregulated m^6^A peaks and 184 downregulated m^6^A peaks (with a fold-change ≥1.5). Further examination of the genomic distribution in all three mRNA regions of the differential m^6^A peaks revealed that most of the differential m^6^A peaks were in CDS and 3′UTR (Supplementary Figure S4A). When we mapped these reads of differential m^6^A peaks to the genome, we identified 1590 genes with upregulated m^6^A peaks and 166 genes with downregulated m^6^A peaks (Supplementary Figure S4B). To further assess the biological significance of dysregulated m^6^A modification in the fetal intestine, we executed GO analysis of differentially m^6^A-methylated genes (Figure 4B and Supplementary Figure S4C). When we concentrated on the functions of m^6^A-hypermethylated genes, we found that they were significantly enriched in the Wnt signaling pathway. The read coverage plot of a representative gene *Wnt4* is shown in Figure 4C. The differential m^6^A levels of representative genes (*Wnt4*, *Fzd5*, *Fzd8*, *Sulf1*, *Sox13*, *Axin2*, and *Abl2*) were confirmed by the MeRIP-qPCR analysis (Figure 4D), and their transcript levels were all attenuated in GFI compared to SPFI as revealed by the RT-qPCR analysis (Figure 4E). This indicates that differential m^6^A modifications in these two types of fetal intestines are correlated with the expression of genes enriched in the Wnt signaling pathways. Next, we knocked out *Mettl3* (*Mettl3* ^*-*/-^) in the mES cell line using CRISPR/Cas9, and we consistently found that *Mettl3* knockout significantly decreased m^6^A levels of representative genes while increasing mRNA expression levels (Figures 4F,G). We further investigated whether the changes in m^6^A methylation would affect mRNA levels of representative genes in mESC. We observed that in the presence of actinomycin D (an inhibitor of mRNA transcription), *Mettl3* knockout retards the degradation of representative genes mRNAs (Figure 4H). Collectively, these data suggest that the maternal microbiome regulates fetal intestinal m^6^A-modified genes in the Wnt signaling pathway.

### Antibiotic Treatment Mostly Recapitulates m^6^A Change in the Mouse Fetal Intestine and Brain

**FIGURE 5:**
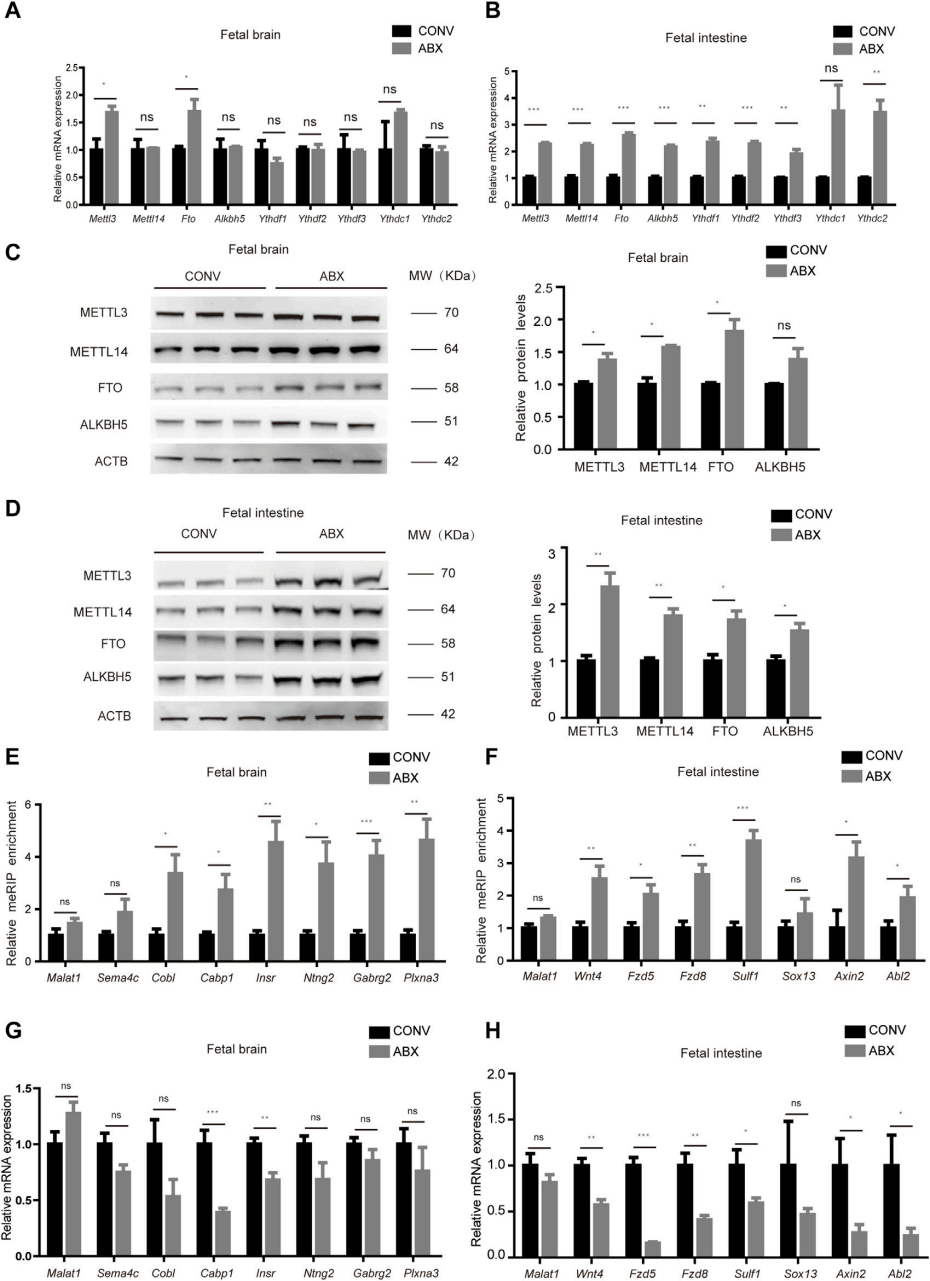
Antibiotic treatment mostly recapitulates m^6^A alterations in the mouse fetal intestine and brain. **(A,B)** Relative mRNA expression levels of m^6^A writers and erasers in CONV and ABX fetal brains **(A)** and intestines **(B)**. **(C,D)** Western blot shows the protein expression levels of m^6^A writers and erasers in fetal brains **(C)** and intestines **(D)** of CONV and ABX mice, and relative protein expression levels were calculated based on the band density in Western blotting results. **(E,G)** Validation of the relative m^6^A enrichment of *Sema4c*, *Cobl*, *Cabp1*, *Insr*, *Ntng2*, *Gabrg2*, and *Plxna3* in CONV and ABX fetal brains **(E)** and of *Wnt4*, *Fzd5*, *Fzd8*, *Sulf1*, *Sox13*, *Axin2*, and *Abl2* in CONV and ABX fetal intestines **(G)** by m^6^A-immunoprecipitation (IP)-qPCR. **(F,H)** Validation of the relative mRNA expression levels of *Sema4c*, *Cobl*, *Cabp1*, *Insr*, *Ntng2*, *Gabrg2*, and *Plxna3* in CONV and ABX fetal brains **(F)** and of *Wnt4*, *Fzd5*, *Fzd8*, *Sulf1*, *Sox13*, *Axin2*, and *Abl2* in CONV and ABX fetal intestines **(H)**.

To confirm the aforementioned results, we treated CONV pregnant mice with a mixture of four antibiotics (vancomycin, neomycin, ampicillin, and amphotericin-B) to mimic germ-free status (ABX mice) and validated that intestinal microbiota were almost exhausted by the 16S rRNA gene qPCR analysis (Supplementary Figure S5A). Similar to our previous experimental results, the mRNA expression levels of *Mettl3* and *Fto* in the ABX fetal brain were slightly higher than those in the CONV fetal brain (Figure 5A), while the mRNA expression levels of both m^6^A writers and erasers in the ABX fetal intestine were significantly increased compared to the CONV fetal intestine (Figure 5B). In addition, the expression of these proteins remained unchanged in fetal livers from both CONV and ABX (Supplementary Figure S5B). As for the protein expression levels of m^6^A writers and erasers, we noted a universal tendency for them to increase in the ABX fetal brain and intestine (Figures 5C,D). We then determined the m^6^A levels and the expression of representative genes regulated by the maternal microbiome in the ABX and CONV fetal brain and intestine, and we found that the m^6^A levels of these genes in the ABX brain and intestine were also increased relative to CONV (Figures 5E,F), and their transcript levels were confirmed by the RT-qPCR analysis (Figures 5G,H). Collectively, these data show that antibiotic treatment mostly recapitulates m^6^A alterations in the mouse fetal intestine and brain.

### Loss of Heterozygosity of *Mettl3* Inhibits the Susceptibility of the Mouse Fetal Intestine to the Maternal Microbiome

**FIGURE 6:**
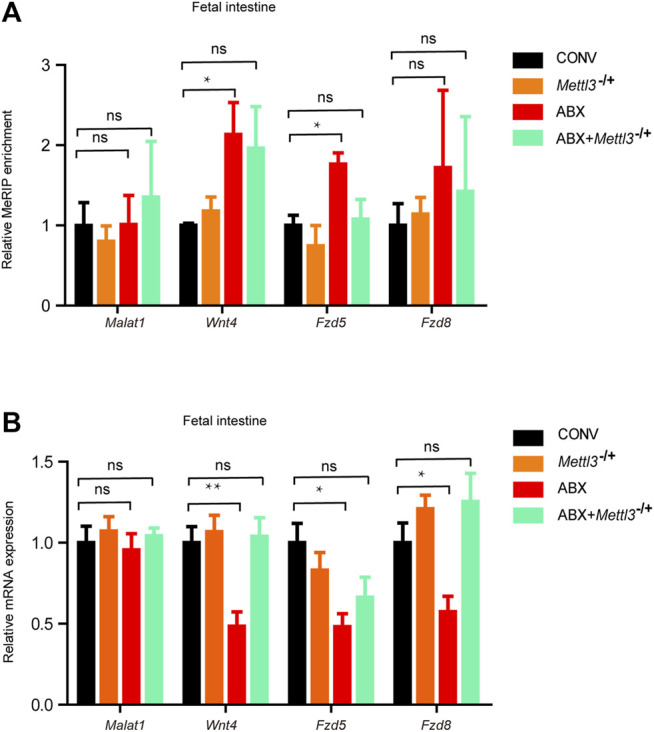
Loss of heterozygosity of *Mettl3* inhibits the susceptibility of the mouse fetal intestine to the maternal microbiome. **(A)** Validation of the relative m^6^A enrichments of *Wnt4*, *Fzd5*, and *Fzd8* in the intestines of CONV, *Mettl3* ^−/+^, ABX, and ABX + *Mettl3* ^−/+^ fetal mice by m^6^A-immunoprecipitation (IP)-qPCR. **(B)** Validation of the relative mRNA expression levels of *Wnt4*, *Fzd5*, and *Fzd8* in the intestines of CONV, *Mettl3* ^−/+^, ABX, and ABX + *Mettl3* ^−/+^ fetal mice.

To further confirm that the expression of developmental genes was regulated by m^6^A as programed by the maternal microbiome, we generated *Mettl3* ^−/+^ heterozygous mice (Supplementary Figures S6A,B). Because the homozygous knockout of *Mettl3* was embryonically lethal, we crossed *Mettl3* heterozygous knockout male mice (*Mettl3* ^−/+^) with wild-type (WT) female mice. The latter were provided with water (i.e., the offspring of CONV and *Mettl3* ^−/+^ mice) or ABX (i.e., the offspring of ABX and ABX + *Mettl3* ^−/+^mice). As expected, there were no significant differences in m^6^A levels of representative genes between CONV and *Mettl3* ^−/+^ fetal intestines, however, m^6^A levels of representative genes increased in the ABX fetal intestine but not in the ABX + *Mettl3* ^−/+^ fetal intestine, compared with the CONV fetal intestine (Figure 6A). Correspondingly, mRNA expression levels of representative genes showed no differences between CONV and *Mettl3* ^−/+^ fetal intestines, while they were significantly reduced in the ABX fetal intestine but not in the ABX + *Mettl3* ^−/+^ fetal intestine, compared with the CONV fetal intestine (Figure 6B). Our collective results, therefore, indicate that the maternal microbiome affects the developmental gene expression via m^6^A modifications.

## Discussion

The microbiome and m^6^A modifications are closely related to human health and disease, and previous studies have shown that host m^6^A is strongly affected by the mouse microbiome (Wang et al., 2019; Jabs et al., 2020). However, the impact of the maternal microbiome on the m^6^A epitranscriptome of the mouse fetus has not yet been elucidated. In this study, we profiled the transcriptome-wide m^6^A methylome of the mouse fetal brain and intestine, demonstrated the alterations in m^6^A methylation caused by the maternal microbiome, identified differential m^6^A peaks, and showed that genes with dysregulated m^6^A peaks were potentially active in fetal development.

In a recent study, Wang et al. (2019)ascertained that both m^6^A writers and erasers were highly overexpressed in the GF brain compared to the SPF brain regardless of RNA and protein levels. Intriguingly, our results also revealed that the maternal microbiome also altered the expression of m^6^A writers and erasers in the mouse fetal brain and intestine, and that expression was recapitulated by antibiotic treatment.

Previous studies have depicted depletion of the maternal microbiome as impairing fetal thalamocortical axonogenesis (Vuong et al., 2020). Our results suggested that loss of the maternal microbiome contributed to m^6^A-hypermethylated genes in GFB, and that these genes were significantly enriched in pathways related to neurodevelopment, including synapse formation and axonogenesis. Moreover, the mRNA expression levels of these m^6^A-hypermethylated genes were commensurately altered, implying that m^6^A plays a key role in effects engendered by the maternal microbiome on fetal neurodevelopment. It is worth noting that increasing evidence reveals a functional link between gut bacteria and neurodegenerative diseases such as Alzheimer’s disease and Parkinson’s diseases (Sampson et al., 2016; Bhattarai et al., 2021; Willyard, 2021), and the abnormality in m^6^A is involved in neurological dysfunction and behavioral defects (Mitropoulos et al., 2017; Chen et al., 2019; Han et al., 2020; Huang et al., 2020). However, although it remains elusive whether the regulation of fetal neurodevelopment by the maternal gestational microbiome increases the risk of neurologic diseases in adulthood, we expect that the elucidation of such a mechanism will provide a foundation for future novel treatments of nerve diseases.

The Wnt signaling pathway is highly conserved from nematodes to mammals (Kohn and Moon, 2005; Clevers and Nusse, 2012) and is involved in many aspects of embryonic development (Clevers, 2006; MacDonald et al., 2009). Current evidence indicates that the Wnt signaling pathway regulates the self-renewal or differentiation of intestinal stem cells (Reya and Clevers, 2005; Böttcher et al., 2021). Our results showed that upregulated m^6^A genes in GFI were enriched in the Wnt signaling pathway but that mRNA expression levels of these genes were downregulated compared with SPFI, the mechanism of which is that m^6^A accelerates the degradation of these genes mRNA. These results reveal that the maternal microbiome regulates the gene expression in Wnt signaling by m^6^A in the fetal intestine, and this may constitute a mechanism whereby loss of the maternal microbiome impairs fetal intestinal development. In addition, maternal exposure to antibiotics promotes gut dysbiosis and increases the risk of inflammatory bowel diseases in offspring (Miyoshi et al., 2017). Although such data suggest that the maternal gestational microbiome exerts a critical effect on the onset and progression of intestinal diseases in offspring, the precise role of m^6^A in this action requires further clarification.

For further verification, we treated conventional mice (CONV) with antibiotics (ABX) to mimic GF status. Although antibiotic treatment mostly recapitulates m^6^A change in mouse fetus, some differences in m^6^A epitranscriptome between GF and ABX mouse still exist. For example, the mRNA expression levels of *Mettl14* and *Alkbh5* were increased in GFB but not in the ABX mouse fetal brain, which may be caused by some individual differences among different mice due to age, nutritional status, or other factors (Jabs et al., 2020). In addition, ABX treatments are unable to completely eradicate the microbiome so there still exists a small amount (about 5%) of the microbiome in ABX mice compared with GF mice, and the acute or subacute aseptic state simulated by antibiotic treatment is not exactly the same as the chronic rearing under an aseptic environment for a long time (Vuong et al., 2020), which may also be some important reasons why the m^6^A epitranscriptome of GF mouse has some difference from the ABX mouse.

Collectively, our data reveal programing of the maternal microbiome on m^6^A modifications in the mouse fetus and should assist in unveiling the underlying mechanisms by which gut dysbiosis precipitates human disease. With progressively maturing analyses and technical developments, we expect that m^6^A will evolve into a potential therapeutic target of microbiota-directed disease.

## Data Availability

The datasets presented in this study can be found in online repositories. The names of the repository and accession number can be found as follows: http://bigd.big.ac.cn/gsa, CRA006146.
