# Corrigendum: The maternal microbiome programs the m^6^A epitranscriptome of the mouse fetal brain and intestine

**DOI:** 10.3389/fcell.2025.1553431

**Published:** 2025-02-06

**Authors:** Zhuoyu Xiao, Sun Liu, Zengguang Li, Jinru Cui, Hailan Wang, Zihan Wang, Qihuan Ren, Laixin Xia, Zhijian Wang, Yuan Li

**Affiliations:** ^1^ Department of Developmental Biology, School of Basic Medical Sciences, Southern Medical University, Guangzhou, China; ^2^ Department of Obstetrics and Gynecology, Nanfang Hospital, Southern Medical University, Guangzhou, China

**Keywords:** maternal microbiome, m^6^A, fetal development, Wnt signaling pathway, METTL3

In the published article, there was an error in the **Funding** statement. The funding statement for the National Key R&D Program of China was incorrectly displayed as “grant nos. 2019YFA802303 and 2021YFA0805400”. The correct statement is “the National Key R&D Program of China (grant nos. 2019YFA0802303 and 2021YFA0805400)”. The correct Funding statement appears below.

