# Supplementary figures and images for: The Maternal Microbiome Programs the m6A Epitranscriptome of the Mouse Fetal Brain and Intestine
Source: Front Cell Dev Biol. 2022 Jul 7;10:882994. doi: 10.3389/fcell.2022.882994 (PMC9301011; doi:10.3389/fcell.2022.882994)

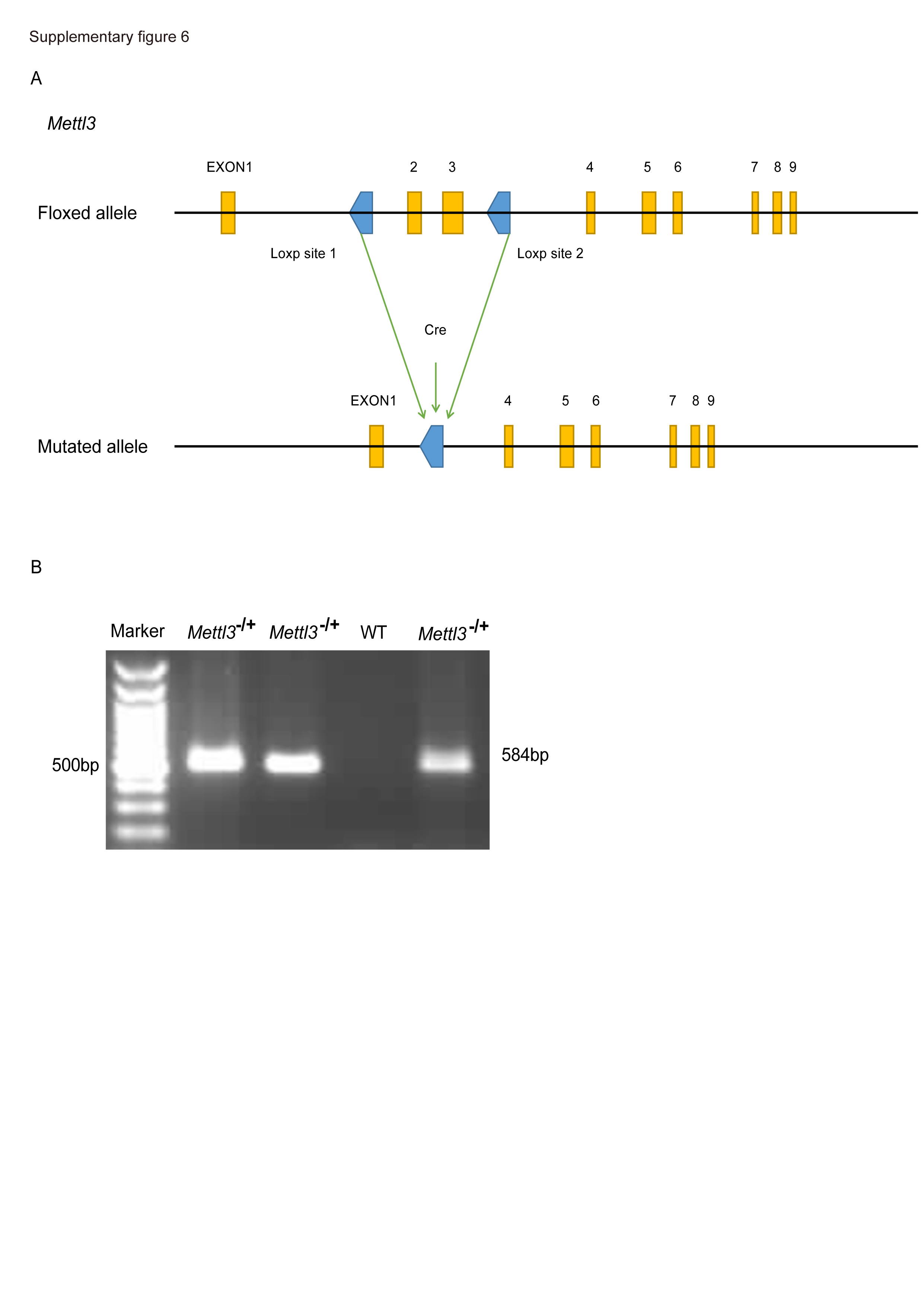

Supplement: Supplementary file 1 [file Image6.TIF]

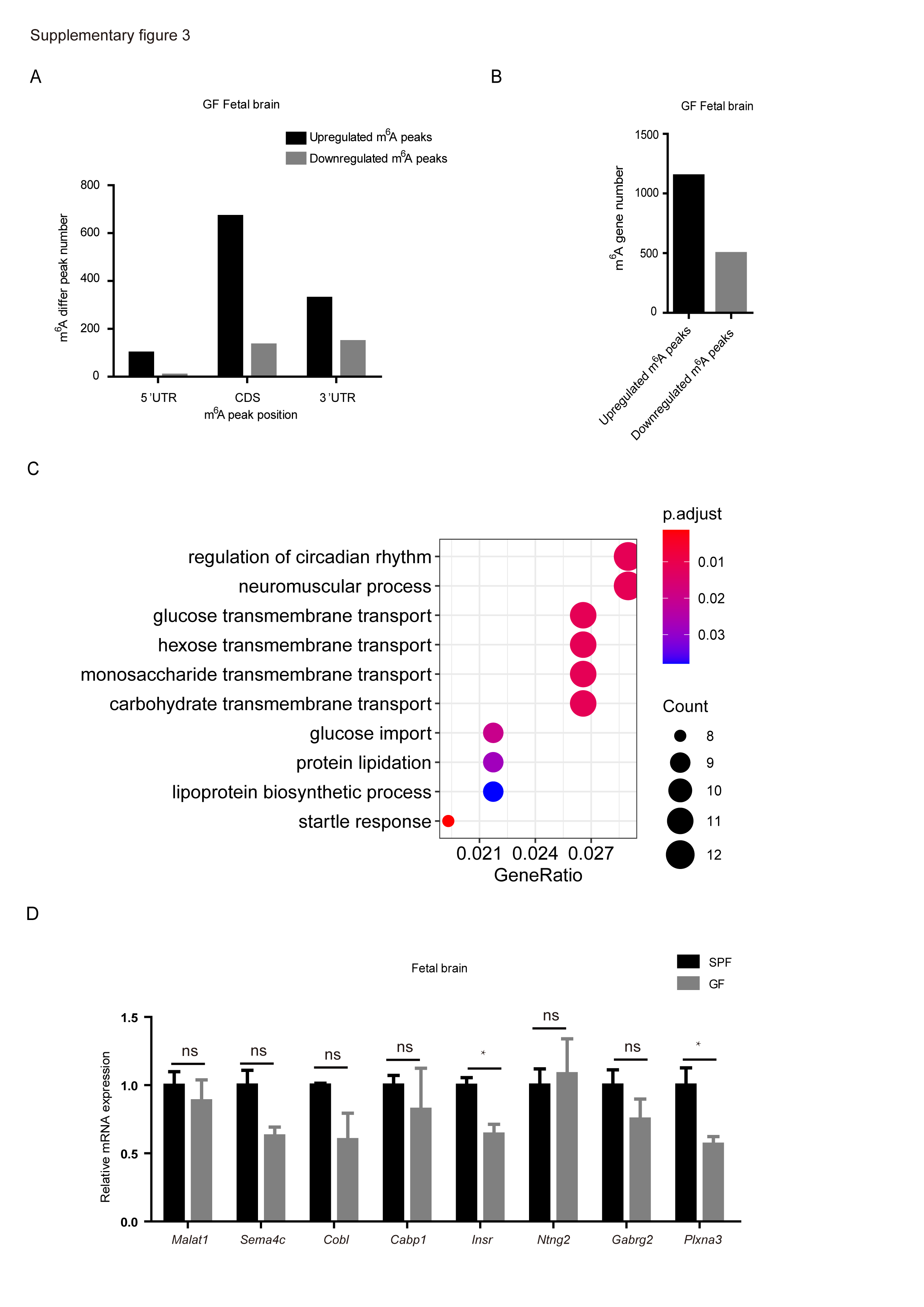

Supplement: Supplementary file 2 [file Image3.TIF]

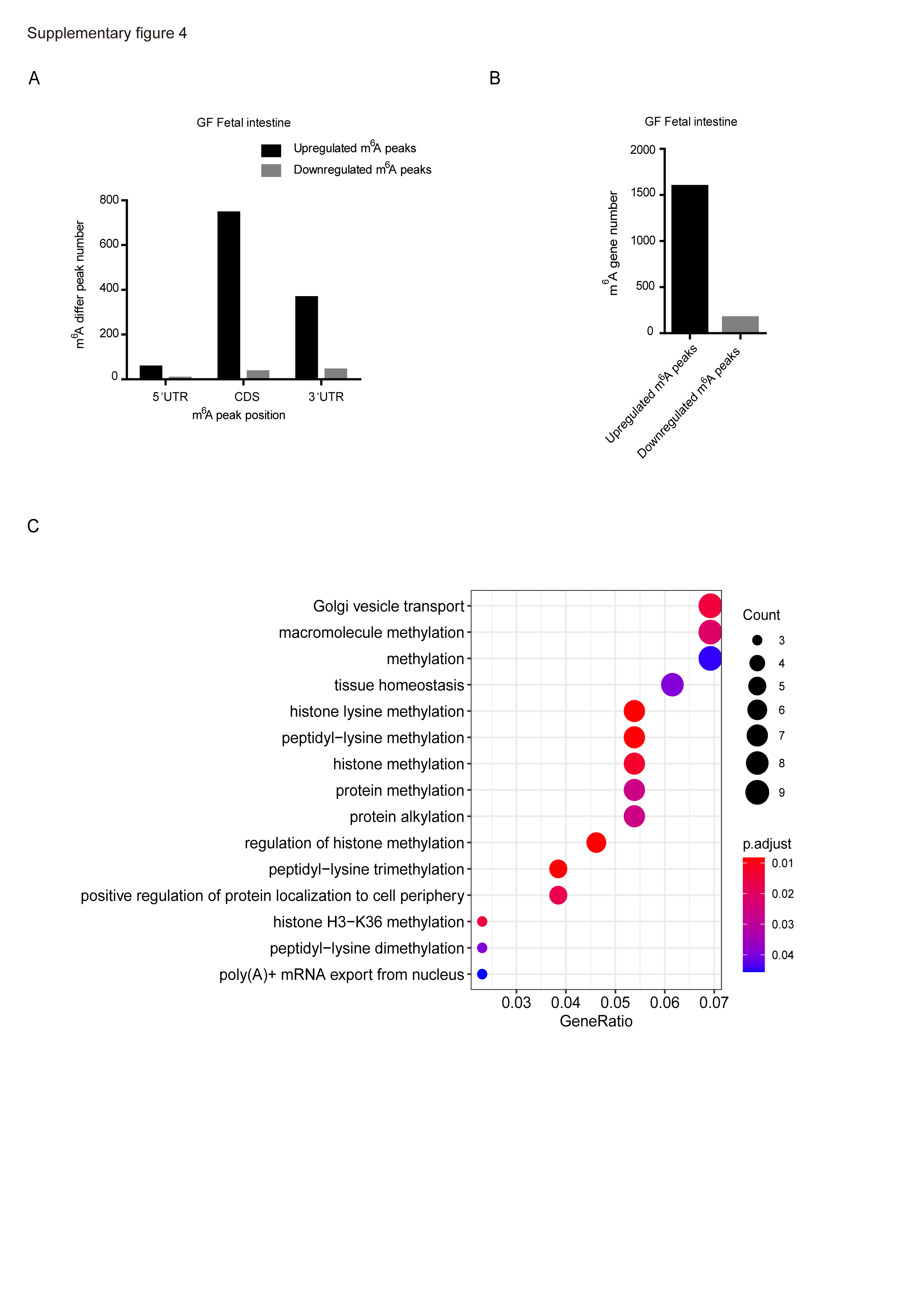

Supplement: Supplementary file 3 [file Image4.TIF]

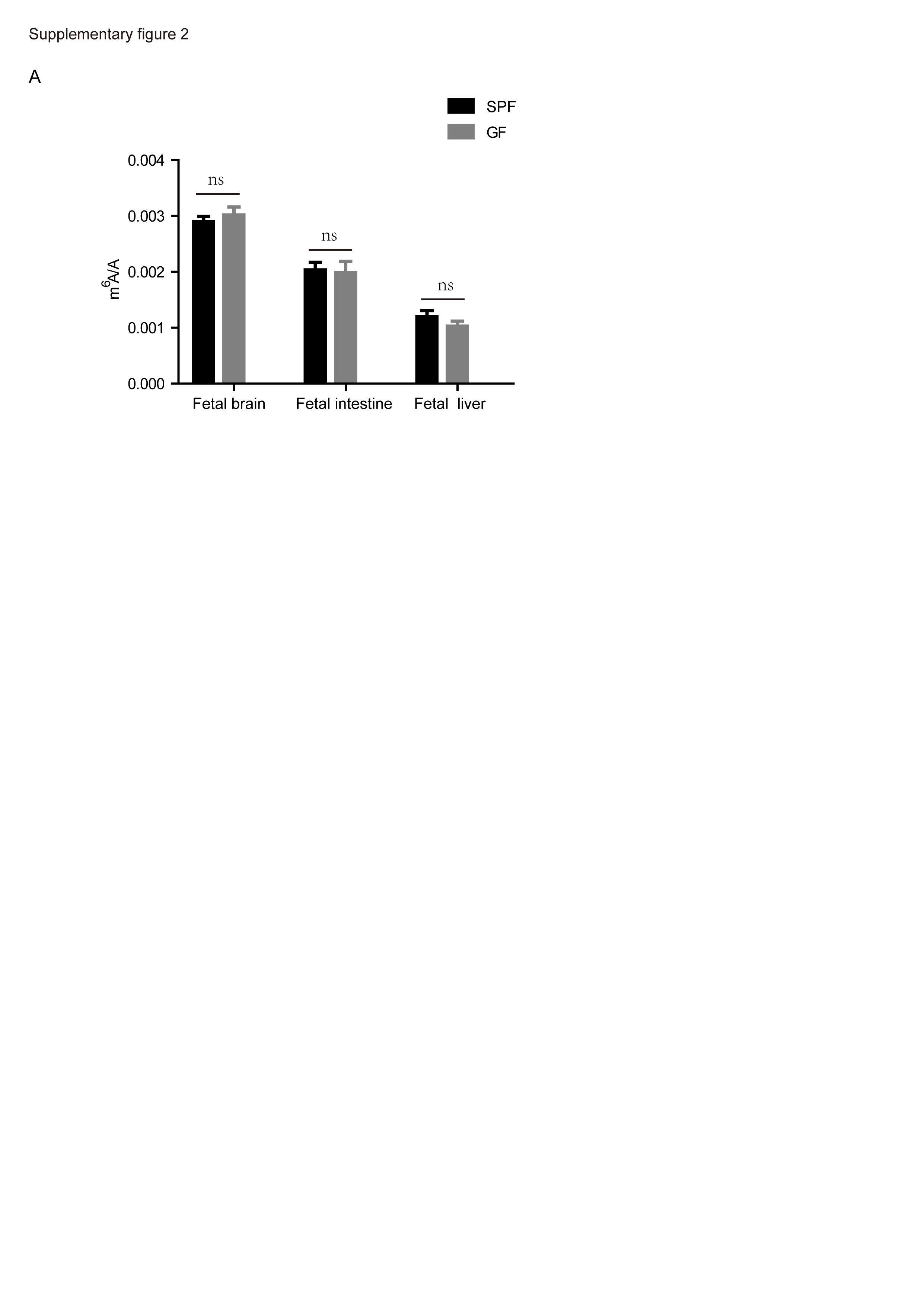

Supplement: Supplementary file 4 [file Image2.TIF]

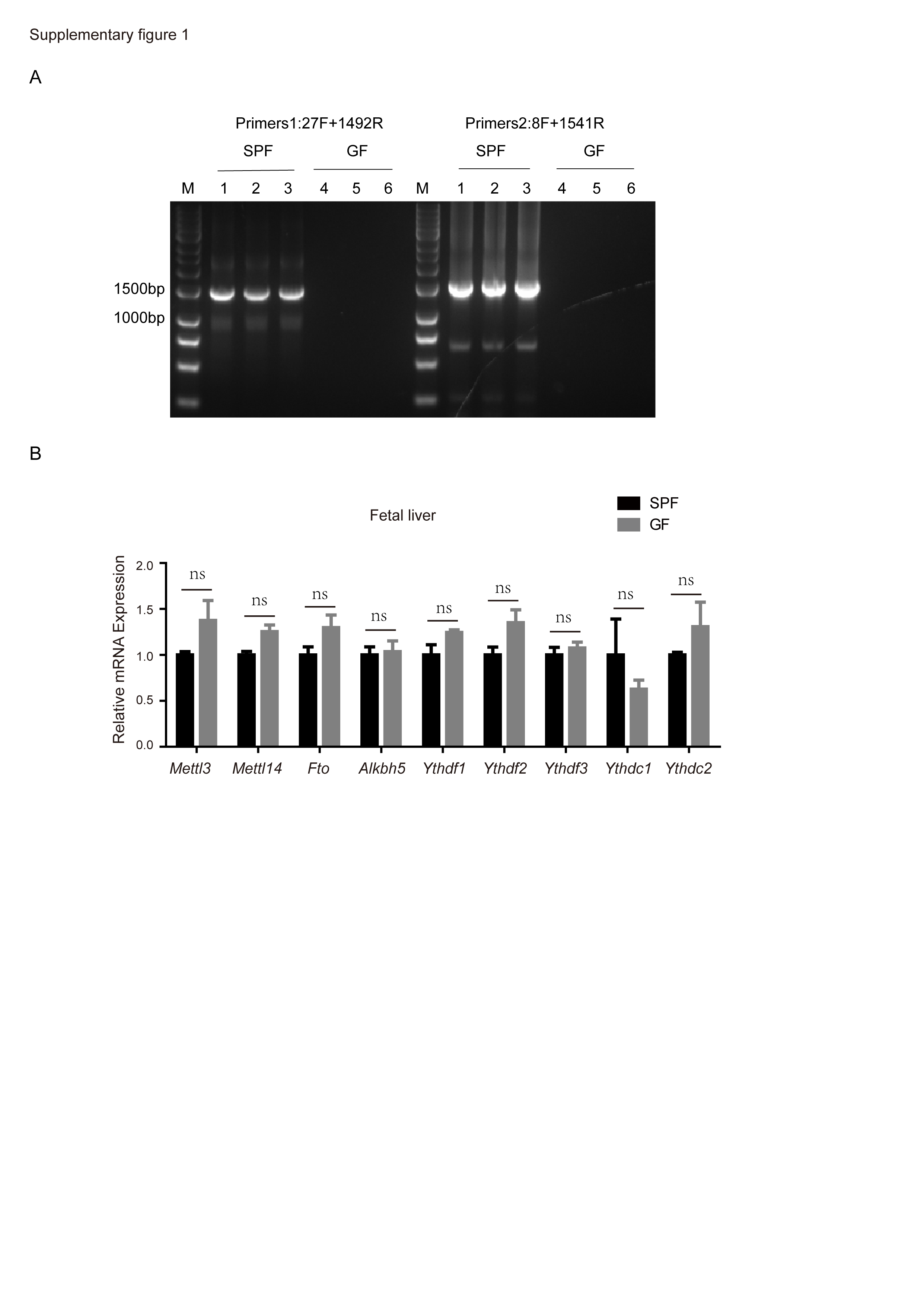

Supplement: Supplementary file 5 [file Image1.TIF]

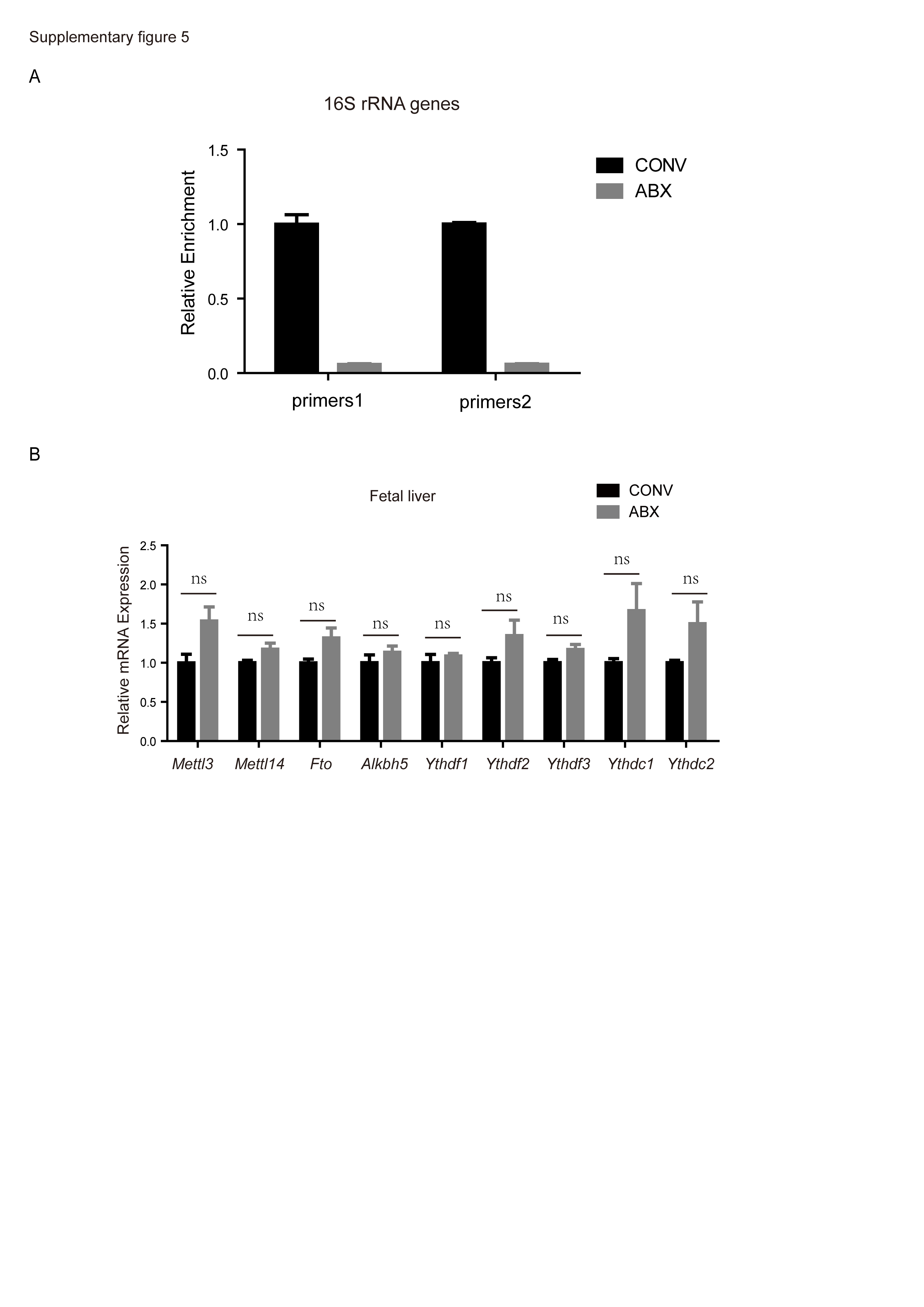

Supplement: Supplementary file 8 [file Image5.TIF]
