# Supplementary material for: The Maternal Microbiome Programs the m6A Epitranscriptome of the Mouse Fetal Brain and Intestine
Source: Front Cell Dev Biol. 2022 Jul 7;10:882994. doi: 10.3389/fcell.2022.882994 (PMC9301011; doi:10.3389/fcell.2022.882994)
Supplement: Supplementary file 6 [file DataSheet1.pdf]

Supplemental Figure 1.

(A) The DNA extracts from the intestinal content from SPF and GF mice were used to 16S rRNA gene PCR amplification with two sets of universal bacterial primers and PCR products were analysed by agarose-gel electrophoresis to confirm the condition of GF mice.

(B) Relative mRNA expression levels of m^6A writers, erasers, and readers in the livers of SPF and GF fetal mice.

Supplemental Figure 2.

(A) LC-MS/MS assay revealing the total m^6A levels in fetal brain, intestine, and liver of SPF and GF fetal mice.

Supplemental Figure 3.

(A) The mRNA positions of differential m^6A peaks in GF fetal brain.

(B) The number of genes containing differential m^6A peaks in GF fetal brain.

(C) Gene ontology-enrichment analysis of genes containing downregulated m^6A peaks in GF fetal brain.

(D) Validation of the relative mRNA expression levels of *Sema4c, Cobl, Cabp1, Insr, Ntng2, Gabrg2,* and *Plxna3* in the fetal brains of SPF and GF mice.

Supplemental Figure 4.

(A) The mRNA positions of differential m^6A peaks in GF fetal intestine.

(B) The number of genes containing differential m^6A peaks in GF fetal intestine.

(C) Gene ontology-enrichment analysis of genes containing downregulated m^6A peaks in GF fetal intestine.

Supplemental Figure 5.

(A) The DNA extracts from the intestinal content from CONV and ABX mice were used for 16S rRNA gene qPCR analysis with two sets of universal bacterial primers to confirm the condition of ABX mice.

(B) Relative mRNA expression levels of m^6A writers and erasers in the liver of CONV and ABX fetal mice.

Supplemental Figure 6.

(A) Targeting strategy used to silence *Mettl3* in mice.

(B) Analysis of PCR products from wild type and *Mettl3*$^{-/+}$ mice by agarose-gel electrophoresis.

Supplementary Table 1. List of utilized primers for 16S rRNA genes detection.

Supplementary Table 2. List of utilized primers for RT-qPCR.

Supplementary Table 3. List of utilized primers for MeRIP-qPCR.

Supplementary Table 4. sgRNA sequence for *Mettl3* knockout.
